# The Right Supramarginal Gyrus Is Important for Proprioception in Healthy and Stroke-Affected Participants: A Functional MRI Study

**DOI:** 10.3389/fneur.2015.00248

**Published:** 2015-12-03

**Authors:** Ettie Ben-Shabat, Thomas A. Matyas, Gaby S. Pell, Amy Brodtmann, Leeanne M. Carey

**Affiliations:** ^1^Neurorehabilitation and Recovery, Stroke, Florey Institute of Neuroscience and Mental Health, Melbourne, VIC, Australia; ^2^Occupational Therapy, School of Allied Health, College of Science, Health and Engineering, La Trobe University, Melbourne, VIC, Australia

**Keywords:** proprioception, kinesthesis, upper extremity, functional laterality, stroke, magnetic resonance imaging, cerebral cortex

## Abstract

Human proprioception is essential for motor control, yet its central processing is still debated. Previous studies of passive movements and illusory vibration have reported inconsistent activation patterns related to proprioception, particularly in high-order sensorimotor cortices. We investigated brain activation specific to proprioception, its laterality, and changes following stroke. Twelve healthy and three stroke-affected individuals with proprioceptive deficits participated. Proprioception was assessed clinically with the Wrist Position Sense Test, and participants underwent functional magnetic resonance imaging scanning. An event-related study design was used, where each proprioceptive stimulus of passive wrist movement was followed by a motor response of mirror ­copying with the other wrist. Left (LWP) and right (RWP) wrist proprioception were tested separately. Laterality indices (LIs) were calculated for the main cortical regions activated during proprioception. We found proprioception-related brain activation in high-order sensorimotor cortices in healthy participants especially in the supramarginal gyrus (SMG LWP *z* = 4.51, RWP *z* = 4.24) and the dorsal premotor cortex (PMd LWP *z* = 4.10, RWP *z* = 3.93). Right hemispheric dominance was observed in the SMG (LI LWP mean 0.41, SD 0.22; RWP 0.29, SD 0.20), and to a lesser degree in the PMd (LI LWP 0.34, SD 0.17; RWP 0.13, SD 0.25). In stroke-affected participants, the main difference in proprioception-related brain activation was reduced laterality in the right SMG. Our findings indicate that the SMG and PMd play a key role in proprioception probably due to their role in spatial processing and motor control, respectively. The findings from stroke-­affected individuals suggest that decreased right SMG function may be associated with decreased proprioception. We recommend that clinicians pay particular attention to the assessment and rehabilitation of proprioception following right hemispheric lesions.

## Introduction

Limb proprioception refers to knowledge of the spatial location of one’s limb in the absence of vision. Proprioception is vital for motor control (1), particularly of the upper limbs (2). It is essential for the control of coordinated movements, especially small or precise movements, and for motor skill acquisition (3). Hence, proprioceptive deficits in the upper limbs are associated with decreased function (1). Despite the importance of proprioception for function, it remains unclear which brain regions beyond the primary sensorimotor cortices (SIMIs) are involved in the processing of proprioception and how this brain activation is altered following focal brain lesions associated with proprioceptive deficits.

Researchers studying brain activation during passive movements of the elbow (4, 5), wrist (6, 7), hand (8), and finger (9, 10) have identified activation in the contralateral primary somatosensory (SI) and motor (MI) cortices and the inferior parietal lobe (IPL). However, investigators disagreed on the pattern (contralateral, ipsilateral, or both) and exact location of activation [supramarginal gyrus (SMG) or the secondary somatosensory cortex (SII)]. In contrast, neurophysiological studies of primates, identified the superior parietal lobe as a key region for the processing of proprioception (11, 12). The ability of current brain imaging paradigms to investigate proprioceptive specific processing, and in particular the contribution from higher order brain regions, requires careful consideration and design.

Inconsistent proprioception-related brain activation has also been reported in high-order motor cortices including the supplementary motor area (SMA), cerebellum (6, 8), and the premotor cortex (PMC) (5, 6, 8). Variations in proprioception-related brain activation may have been due to the fact that brain imaging studies of passive movements varied in paradigm design. In some cases, the support of the moving limb was suboptimal and may have introduced significant tactile stimulation (6, 8, 10), thus generating confounding brain activation.

Proprioception-related brain activation has also been studied using illusory vibrations. This is vibration of a tendon at a frequency between 70 and 100Hz, which creates an illusion of movement (13). Early findings from illusory vibration studies emphasized activation in motor cortices including: MI, SMA, PMC, and the cingulate motor area (14, 15). Later, researchers also identified brain activation in the IPL (5, 16–18). However, as was the case with passive movements, reported activation varied in location, with reports of activation in the parietal operculum (5, 15, 17) or the SMG (16, 18). Hemispheric bias was also controversial with some researchers reporting bilateral activation (16, 18), while others report a right hemisphere dominance (15, 17).

Illusory vibrations provide different peripheral stimuli to passive movements. The stimulus is large phasic and of uniform frequency in the primary afferent fibers of the muscle spindles (19, 20). Minimal, if any, stimulation is produced in the secondary fibers of the muscle spindles and the joint receptors (19, 20). In contrast, passive movements produce multifrequency phasic and tonic stimulation of the primary afferent fibers in the muscle spindles (21). Secondary fibers of the muscle spindles and joint receptors are also stimulated (21–23). It is possible that different peripheral stimuli were associated with differential brain activation (5). In such circumstances, brain activation during passive movements is likely to reflect the central processing of proprioception more accurately than illusory vibration.

An important limitation of both passive movement and illusory vibration brain imaging studies of proprioception is that participants were not required to provide accurate and measurable responses to the proprioceptive stimuli during scanning. Responses to proprioceptive stimuli are important for two reasons. First, by asking participants for accurate responses to proprioceptive stimuli (and monitoring the responses), examiners ensure that participants adequately engage in proprioceptive information processing. Second, the response requirement introduces a certain degree of difficulty to the proprioceptive task, which would not have been present if responses were not required. Increased task difficulty is desirable due to the associated increase in cortical activation (24, 25).

In healthy participants, findings from behavioral studies have suggested asymmetry in the accuracy of proprioception from the right and left limbs (26–28). Asymmetry in behavioral measures suggests hemispheric dominance and thus asymmetry in proprioception-related brain activation. Brain activation studies of illusory vibration stimulation confirmed right hemispheric dominance (15, 17, 18). Brain activation in the IPL and inferior frontal gyrus was found in all three studies, but the exact loci of activation and degree of laterality (i.e., right hemispheric or bilateral activation) varied. None of the brain imaging studies of passive movements investigated laterality of proprioception.

Quantitative behavioral measures of proprioception in stroke-affected individuals have shown deficits in about 50% of the participants (1, 29). Considering the adverse effect of proprioceptive deficits on function (1), it is important not only to understand the central processing of proprioception in healthy participants but also how it changes following brain lesions associated with proprioceptive deficits. This is because proprioception can be rehabilitated (30–32) with associated changes in brain activation (33) and improvement in function (34).

The current study was designed to investigate the brain–behavior relationship of proprioception. The research questions were:

(1) Which high-order brain areas are important for early coding of natural proprioceptive stimuli?
(2) Is proprioception-related brain activation lateralized, and if so in which areas?
(3) How does proprioception-related brain activation in stroke-affected individuals with proprioceptive deficits differ from that of healthy participants?

To answer these questions, we designed an event-related functional magnetic resonance imaging (fMRI) study with a controlled proprioceptive stimulus and response paradigm. The study was exploratory with data-driven laterality analyses.

First, proprioceptive stimuli were delivered with maximal limb support and minimal tactile stimulation to eliminate confounding brain activation. Second, participants were required to respond accurately to each proprioceptive stimulus for optimal brain activation related to attended proprioceptive information processing. Third, the paradigm and analyses were designed to show brain activation at the beginning of a proprioception task during the coding of proprioceptive stimuli. We hypothesized that coding proprioception would involve high-order somatosensory cortices in the parietal lobe including the IPL, the SII, and the superior parietal lobe. We also hypothesized that proprioception-related brain activation would be found in high-order motor cortices in the frontal lobe including the PMC, SMA, and cingulate motor cortex. The second hypothesis was that proprioception-related brain activation would be lateralized to the right hemisphere, particularly the high-order cortices. Finally, we hypothesized that laterality would decrease following stroke which affected proprioception.

## Materials and Methods

### Participants

Twelve healthy right-handed participants (35) were recruited. Participants were aged 23.4 ± 3.3 years (seven females) and their age was restricted (18–30 years) to control for age-related variations in proprioception (36) and brain activation (37). Participants’ proprioception was within the normative range (average absolute error below 11 ± 4.8°) as verified behaviorally with the Wrist Position Sense Test (38).

Three participants with chronic strokes (CSs) and proprioceptive deficits were also recruited: CS1 45 years, male, 16 months post right hemisphere stroke, average absolute wrist position error on the Wrist Position Sense Test was 25.6 ± 22.5°; CS2 65 years, female, 72 months post left hemisphere stroke, average absolute error 17.9 ± 15.2°; and CS3 46 years, male, 68 months post left hemisphere stroke, average absolute error 20.8 ± 18.4°.

Participants had no history of wrist injury, neurological injury (other than the three participants affected by stroke), psychiatric conditions, ongoing medical issues, diabetes, hearing impairments, or any of the standard contraindications to MRI scanning. The study was approved by the La Trobe University and Austin Health Human Ethics Committees, conforming to Declaration of Helsinki standards. Participants gave written informed consent prior to recruitment.

### Experimental Design and Analysis Approach

Participants performed a limb position matching task in the scanner using an event-related study design. The experimental paradigm was carefully constructed to ensure that fMRI data were collected specifically during coding of proprioception and not during response generation. Care was also taken to ensure that other confounding stimuli were excluded. We used an exploratory approach to identify the parietal and frontal regions activated specifically at the beginning of the proprioceptive stimuli during coding of proprioception. Brain laterality analyses were data driven, and only regions that showed significant activation during coding of proprioception were then analyzed for laterality. *A priori* selection of specific brain regions for the laterality analyses was not possible due to the conflicting literature. Testing and analysis of right wrist proprioception and its laterality were performed separately to that of the left wrist. No direct comparisons were made between left and right wrist data. Data of stroke-affected individuals were analyzed as case studies and no direct comparisons were made with data of healthy participants.

### Experimental Paradigm

**Figure 1:**
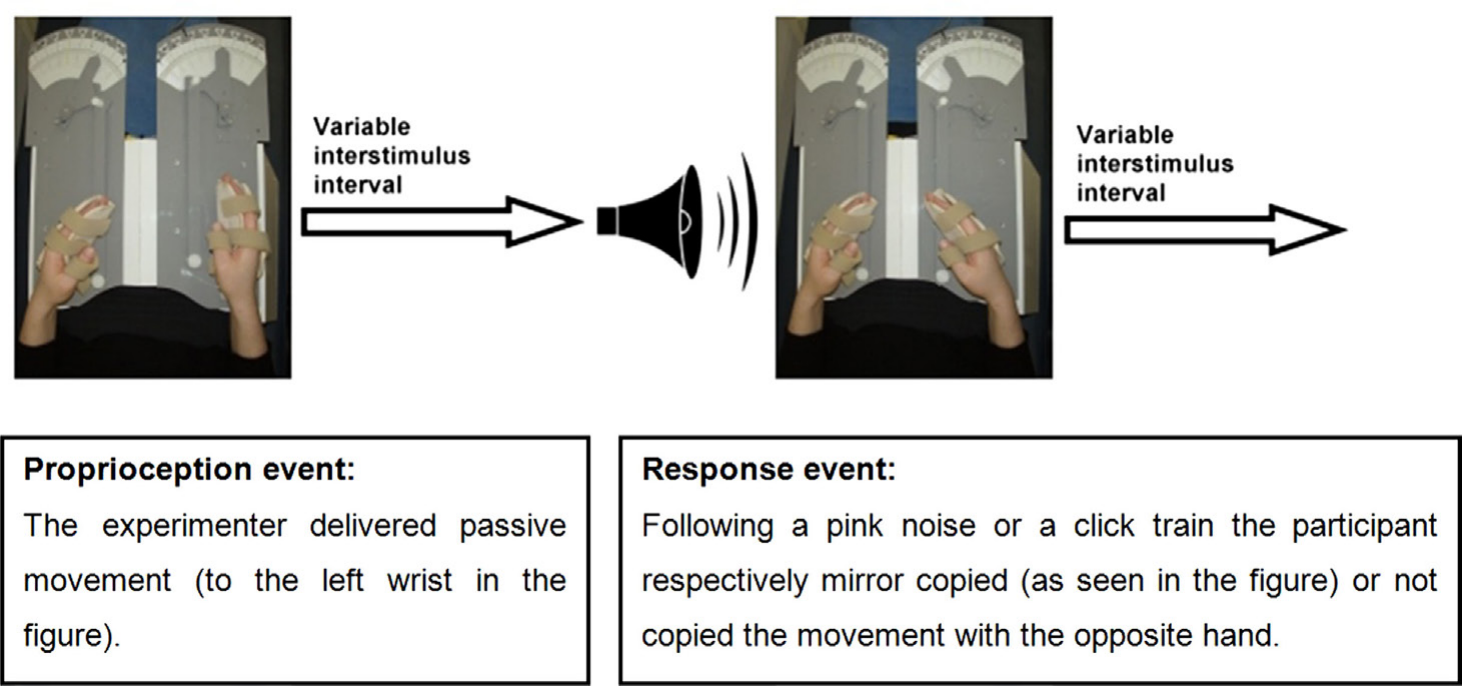
**The event-related experimental design**.

An event-related fMRI study was conducted in which participants performed a limb position matching task. The proprioceptive task was performed with eyes closed to eliminate the effect of vision on proprioception. Participants’ hands were placed in splints attached to a lap-tray (wrist and splint axes were aligned), and their arms were supported on contoured foam cushions. Hand placement was designed to minimize confounding tactile stimulation or voluntary movement. The event-related design enabled temporal separation of brain activation related to proprioception from that related to motor response. A single trial was composed of two events: a proprioceptive stimulus event and a response event (see Figure 1). Each event was followed by a randomly varying interstimulus interval which varied between 0.5 and 12 s: 0.5–6.0 s for 70% of events, 6.0–10.0 s for 20% of events, or 10.0–12.0 s for 10% of events (i.e., jittering) (39). The purpose of the response events was to ensure participants’ vigilance. Hence, the specific pattern of brain activation during response events was not relevant to the research question. The brain activation of interest took place at the beginning of the proprioceptive events, during coding of proprioception.

The investigator was visually cued to passively move the participant’s hand via a lever (to minimize tactile stimulation) for a maximal duration of 3 s. In addition, the investigator was pretrained to deliver passive wrist movements at a rate of 10° a second or faster, to ensure stimulation of the main proprioceptors which are sensitive to changes in joint position, and to produce a phasic firing pattern (40). Passive movements of the wrist were presented in random order to any one of 21 predetermined positions within a 100° range of wrist flexion-extension movements. Positions were analyzed together rather than individually as the research question pertained to proprioception-related brain activation in general and not the differential processing of each position.

Response requirements were designed to ensure maximal attendance to the proprioceptive stimulus. Response events commenced with a 600 ms auditory cue of either a pink noise (random noise with an equal energy in all octaves) or a click train, and participants were allowed 3 s for their response. The pink noise cued the participant to mirror copy the wrist position with the opposite hand (70% of the events), while the click train cued participants not to copy the wrist position (30% of the events). The examiner closely monitored participants’ responses during the scans and accuracy of response measurements were collected in the prescan testing. Vigilance was also monitored in the prescan testing by assessing adherence to auditory sounds that served as cues to either respond or not respond to the proprioceptive stimuli. Responses were considered non-vigilant if participants moved their response hand half way or more toward mirror copying the stimulus position when cued not to respond. Vigilance was scored as percentage of correct adherence to “do not respond” cues. Participants were first studied during left wrist proprioceptive stimuli (LWP) with right wrist responses, and then 2–6 months later during right wrist proprioceptive stimuli (RWP) and left wrist responses. The time between LWP and RWP scans was not expected to affect the results as no direct comparisons were made between the two.

### Tests and Prescan Training Performed Outside the Scanner

The proprioceptive paradigm was practiced in a prescan session, 1–9 days before the scan to ensure familiarity with the task. During the prescan sessions, measurements of angular wrist displacements were taken by potentiometers attached to the wrist axes. Following familiarization, participants’ responses were measured for accuracy and vigilance.

Electromyographic recordings were taken outside the scanner only. The EMG amplifier that we used is designed to work in the electrically noisy clinical environment and therefore has an operating bandwidth of 18–370 Hz. Outside the bandwidth, signal was filtered below −3 db. Notch filter was set at 50 Hz. Rectified signal was then sampled at 10 Hz, and these samples were employed to compute the average signal for each condition: passive movements, active movements, and rest. Recordings were collected simultaneously from two channels (wrist flexors and extensors) during random 30 s blocks of passive movements, active movements, and rest. Recordings were collected over 6.5 min, and 2 s of data was trimmed from the beginning and end of each block to avoid contamination of the data. Data were then normalized in the following manner. For each participant, the median of active movement readings was multiplied by a constant that gave it the value of 100. Then all recordings from the same muscle group were multiplied by this constant. Data of all participants were then pooled, and a non-parametric Wilcoxon *T*-test was conducted to compare EMG recordings during passive and active movements. Statistically significant difference was interpreted as evidence of participants’ ability to relax their forearm muscles during passive movements. This ensured that brain activation was not related to voluntary muscle contraction.

### Data Acquisition

A scanning session contained four runs. Each run extended over 20 trials. Runs commenced with auditory instructions, which lasted for 27 s. The first 12 volumes of each run were discarded (nine volumes of instruction and three equilibration volumes). One hundred and thirty-one whole brain volumes were collected from each run. The computer program Presentation^®^ (Version 9.70^1^) was used to coordinate scanner timing with the delivery times of the visual cues to the investigator and the auditory cues to the participants. The same software served to generate log-files, which recorded event times in each run.

Data were acquired on a 3 T GE Horizon LX MRI scanner (GE Systems, Milwaukee, WI, USA). Tilted axial slices were oriented parallel to a line passing inferior to the genu of the corpus callosum and superior to the cerebellum. The tilted imaging plane served to maximize the signal from the parietal cortex. Functional scans were acquired using a T2*-weighted gradient echo echo-planar imaging sequence [imaging parameters: repetition time = 3000 ms, echo time = 40 ms, flip angle = 75°, field of view = 240 mm, matrix = 128 × 128, 25 slices, 4 mm thick, and 1 mm gap (in-plane resolution 1.875 mm × 1.875 mm)].

Anatomical axial 3D scans were acquired using a T1-weighted FSPGR imaging sequence [repetition time = 13.8 ms, echo time = 2.7 ms, inversion time = 500 ms, flip angle = 20°, field of view = 240 mm, matrix = 512 × 512, 80 slices, 2 mm thick (in-plane resolution 0.47 mm × 0.47 mm)]. Axial 2D T2-weighted image was also taken [repetition time = 3400 ms, echo time = 77 ms, inversion time = 500 ms, flip angle = 90°, field of view = 240 mm, matrix = 512 × 512, 25 slices, 4 mm thick, 1 mm gap (in-plane resolution 0.47 mm × 0.47 mm)].

### Stroke Lesion Mapping

Lesion sites were identified on the non-normalized anatomical axial 3D T1 images of each stroke-affected participant. A neurologist visually mapped the lesion sites to normalized generic axial slices (41) taken from the Talairach atlas (42). A second neurologist then evaluated that the lesions were accurately mapped. While lesion mapping has a subjective element, this process minimized the risk of bias.

### Data Analysis of fMRI Scans

#### Individual Image Processing

Data analyses were carried out using SPM 2 (Wellcome Department of Imaging Neuroscience, London, UK). Raw images were inspected for artifacts or structural abnormalities and then pre-processed: (i) correction for slice acquisition time, (ii) realignment to a target volume closest to the median value of head motion (iBrain™ Version 3^2^ used for median image calculation), (iii) coregistration of anatomical scans to functional scans, (iv) spatial normalization into the Montreal Neurological Institute space [with masking the lesion sites for the stroke-affected participants – cost function masking (43)], and (v) spatial smoothing with a kernel size of 8 mm.

#### Statistical Analyses

Only the beginning of each proprioception event was modeled as the research question was related to brain activation during coding of proprioceptive stimuli. Timing of each event was entered according to time recorded in the Presentation^®^ log-file. We used a hemodynamic response function and included an additional dispersion regressor to allow for the longer event durations in this study (up to 3 s).

It was expected that the brain regions most significantly activated during the beginning of the proprioceptive stimuli (coding of proprioception) would not be activated to the same degree during other components of each trial, namely: response generation, auditory cues, and interstimulus intervals. Therefore, contrasts were generated to identify brain activation that took place at the beginning of proprioception events above conditions of no interest (response generation, auditory cues, and interstimulus intervals). Individual data of healthy participants were analyzed using a standard unpaired *t*-test. The voxel-height threshold was set at *p* < 0.001, uncorrected for multiple comparisons. Analysis at the individual level was exploratory; therefore, a low threshold was selected to reveal trends of brain activation. The threshold used for data of stroke-affected participants was set at *p* < 0.05 corrected for multiple comparisons due to the expected bilateral brain activation (44, 45) of greater extent (44) compared to healthy participants. A high pass filter was used to remove the effect of low frequency drift on the data.

#### Group Analyses

Random effect analyses were used to generate *t*-contrasts for group activation maps of the LWP and RWP scans. As with individual analyses, only the beginnings of proprioception events were modeled, and they were contrasted against all other brain activation that took place during the experiment (response events, auditory cues, and interstimulus intervals). To avoid the risks of multiple comparisons, cluster correction (minimum cluster size of 20 voxels) for multiple comparisons was used at *p* < 0.05 (contrasts entered in the analysis were at voxel-height threshold of *p* < 0.001). Anatomical loci of significant activation were identified using probabilistic maps (46) available from the SPM2 toolbox.

**Figure 2:**
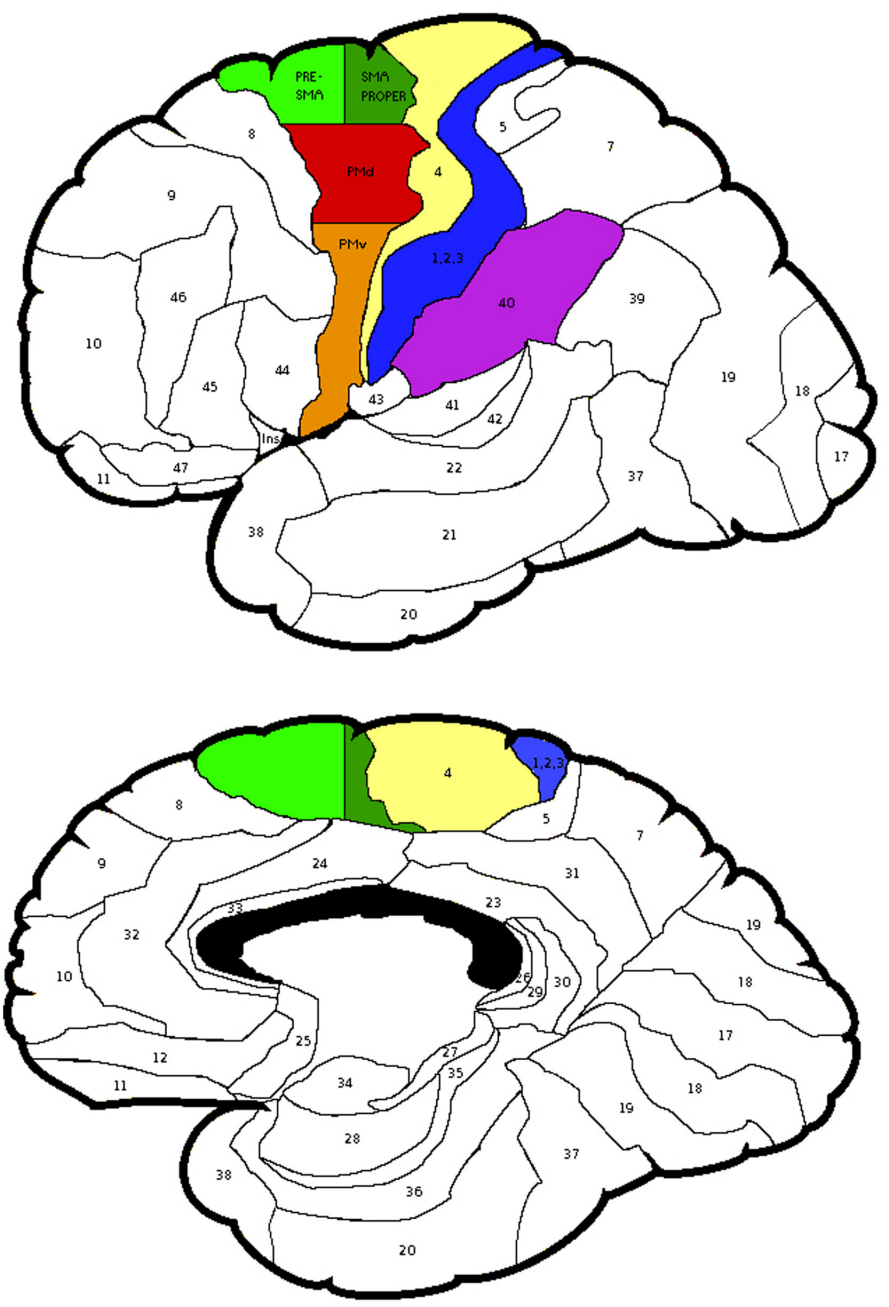
**The regions of interest selected for the laterality calculations and the subdivisions of Brodmann Area 6**. Areas depicted: Brodmann Areas 1,2,3, primary somatosensory cortex; area 4, primary motor cortex; area 40, supramarginal gyrus; PMv, ventral premotor cortex; PMd, dorsal premotor cortex; subdivisions of area 6, Pre-SMA, pre-supplementary motor area and SMA proper, supplementary motor area proper.

The probabilistic maps, however, did not specify the cytoarchitectonic probability of Brodmann area (BA) 6. Thus, using the Talairach coordinates BA 6 was divided into lateral and medial parts. The area lateral to *x* = 15 was considered as the PMC and medial to it, the SMA. The PMC was divided into superior and inferior areas. The area superior to *z* = 42 was considered as the dorsal PMC (PMd), while inferior to it was the ventral PMC (PMv). The SMA was divided into anterior and posterior parts. The area anterior to *y* = 0 was considered as pre-SMA, while posterior to it was interpreted as the SMA proper [see Figure 2, (47)].

#### Laterality Analyses

Laterality calculations in the form of laterality index (LI) were used to quantify the hemispheric symmetries of proprioception-related brain activation during LWP and RWP separately, and no direct comparisons were made between the two. Anatomical brain regions selected for the LI calculations (regions of interest) were the primary SI and MI (based on the literature reviewed in Section “Introduction”), and more importantly high-order somatosensory and motor cortices identified in both the LWP and RWP group analyses. Outlines for the regions of interest were defined using an independent template – the Wake Forest University PickAtlas available from the SPM2 toolbox. For BA 6, outlines of subregions were generated manually using the FSLView tool (Version 3.0), in accordance with the guidelines detailed in Section “Group Analyses.”

Laterality was determined using signal extent based on the previously described protocol (48). Signal intensity of each voxel in the region of interest was determined by the statistical parametric maps of the LWP and the RWP contrasts. The average signal intensity was then calculated for the 5% of voxels showing the highest *t*-score. The LI was calculated as: (right − left)/(right + left). Using the top 5% of voxels showing the highest *t*-score served to reduce the risk of confounding brain activation related to inhomogeneities in the magnetic field or multiple comparisons. This risk was also reduced by contrasting brain activation during proprioceptive coding with all other experimental conditions (response generation, auditory processing, and rest), rather than contrasting with rest only.

Laterality thresholding is designed to limit type I errors. Based on the literature, we selected an *a priori* threshold of −0.2 ≥ LI value ≥ 0.2 to indicate lateralized brain function (49). Thus, we expected that in the dominant region the area of the most significant brain activation showed at least 33% higher signal intensity compared to the homologous area. LIs were calculated for each ROI of each participant based on the individual analyses. Group LIs were reported as mean and standard deviation.

## Results

### Clinical and Proprioception Results

All healthy participants completed the LWP scans and six completed the RWP scans. The other six were not available to participate in the RWP study. During the prescan sessions, participants were vigilant for 96.8% of the tested trials (range: 89–100%, SD = 4.8%). The mean absolute error of participants’ response accuracy for the matching task performed in the scanner was 8.6° (SD = 2.7°) for LWP and 7.5° (SD = 0.9°) for RWP.

As with the previous studies (5), forearm muscle electromyographic recordings for healthy participants during passive movements (mean 10.73, SD 7.70) were significantly lower than during active movements (116.96, 76.44) when tested with the Wilcoxon *T* test (*p* < 0.001).

**Figure 3:**
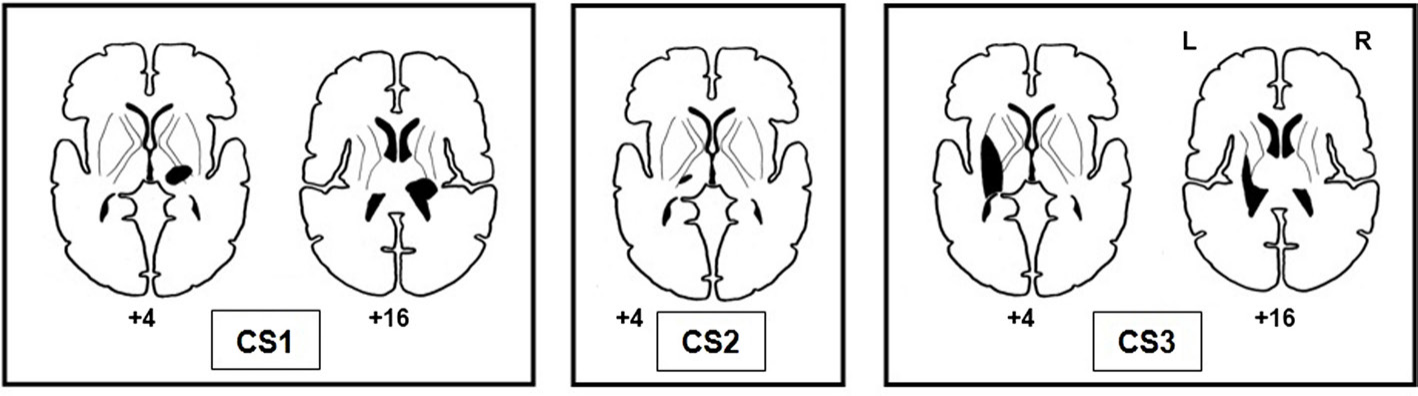
**Lesion sites of the three stroke-affected participants**.

Lesion sites of stroke-affected participants were subcortical, and the common lesion site was the thalamus (see Figure 3). The lesions of CS1 and CS3 extended to include the posterior limb of the internal capsule and the basal ganglia. During the prescan session, the mean absolute error of response for CS1 (LWP) was 17.9° (SD = 9.6°), vigilance 91.7%; for CS2 (RWP) mean absolute error of response 7.5° (SD = 7.0°), vigilance 94.4%; and for CS3 (RWP) mean absolute error of response 19.6° (SD = 13.3°), vigilance 100%.

### Cortical Areas Activated During Proprioception

**Figure 4:**
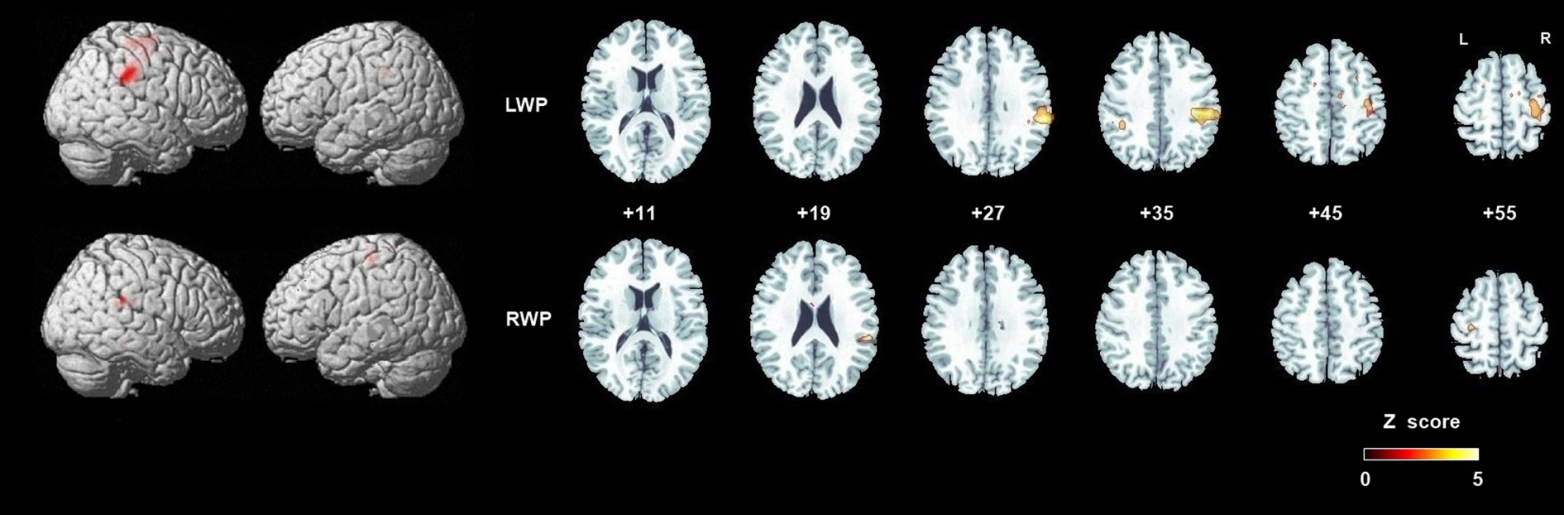
**Group analyses of brain activation in healthy participants during proprioception**. Group brain activation was overlaid on a whole brain and axial sections of the Montreal Neurological Institute template. Threshold level *p* < 0.05 corrected at the cluster level. Abbreviations: LWP, left wrist proprioception; RWP, right wrist proprioception.

Group brain activation of healthy participants during the LWP task was in the right SI cortex, particularly in BA 3a, the right SMG, PMd, MI (BA 4a and 4p), superior and middle frontal gyri, SMA proper, and the middle cingulate cortex (see Table 1; Figure 4). Group brain activation during performance of the RWP task was significant in the right SMG, the left PMd, and MI (BA 4a) (see Table 1; Figure 4).

**Table 1:** **Group analyses of brain activation loci in healthy participants during proprioception**.

Task	Anatomical location	BA	Cluster size	*Z* score	Talairach coordinates		
					*x*	*y*	*z*
LWP	R SI	3a	844	4.57	34	−32	45
	R SMG^a^	40		4.51	52	−40	37
	R PMd^a^	6		4.10	32	−26	69
	R MI	4a		3.96	36	−32	69
	R MI	4p		3.86	36	−22	53
	R SFG^a^	6/8		3.32	24	4	57
	R MFG	6/8		3.31	26	6	53
	R SMA (proper)^a^	6	83	3.75	16	−12	61
	R MCC^a^	6/24		3.19	10	−8	49

RWP	R SMG^a^	40	33	4.24	56	−38	29
	L PMd	6	29	3.93	−32	−26	64
	L MI^a^	4a		3.38	−36	−32	69

*Clusters of proprioception-related brain activation are reported at the cluster-level threshold of *p* < 0.05 FDR corrected*.

*^a^Anatomical locations of more than one maxima. Within each cluster (>20 voxels), only the most significant maximum is listed per anatomical location. BA, Brodmann area; L, left; LWP, left wrist proprioception; PMd, dorsal premotor cortex; R, right; RWP, right wrist proprioception; SFG, superior frontal gyrus; SI, primary somatosensory cortex; SMA, supplementary motor area; SMG, supramarginal gyrus; SPL, superior parietal lobe; MCC, middle cingulate cortex; MI, primary motor cortex; MFG, middle frontal gyrus*.

Proprioception-related brain activation varied among stroke-affected participants; however, common areas of brain activated included the IPL, SPL, and PMd (see Table 2).

**Table 2:** **Individual brain activation loci of stroke-affected participants during proprioception**.

Participant and task	Anatomical location	BA	Cluster size	*Z* score	Talairach coordinates		
					*x*	*y*	*z*
CS1 LWP	L IPL^a^	40/7	272	7.44	−42	−50	53
	L Sup M Gyr^a^	6	182	7.26	−4	22	53
	L SMA (proper)	6		6.99	−4	16	61
	R ITG	37	58	7.22	58	−60	−7
	R SPL	7	111	6.99	16	−72	65
	R IPL^a^	40	149	6.96	40	−54	53
	R SMG^a^	40	126	5.94	56	−38	33
	L PMd^a^	6	87	5.91	−36	−8	65

CS2 RWP	L SPL^a^	7	458	7.26	−26	−56	73
	L SI^a^	2		7.19	−34	−40	57
	L SI^a^	1		6.64	−36	−42	73
	L IPL^a^	40	292	7.15	−54	−40	45
	L STG	41/42		5.76	−64	−42	25
	L SMG	40		5.50	−54	−48	29
	L PMd^a^	6	130	6.37	−24	−20	81
	L SMA (proper)	6		5.99	−6	−14	73
	R SMG^a^	40	80	5.74	56	−46	49
	R IPL^a^	40		5.06	58	−34	57

CS3 RWP	L PMd^a^	6	340	Inf	−32	−14	73
	L MFG	6		4.95	−24	−4	61
	R IPL^a^	40	519	Inf	32	−54	45
	R SMG^a^	40		6.50	40	−38	45
	R SPL^a^	7	421	Inf	12	−86	57
	R cuneus	18/19		Inf	12	−88	49
	L SOG	18		7.19	−10	−88	45
	L cuneus^a^	18		5.11	−6	−98	25
	L SMG^a^	40	304	Inf	−66	−38	37
	L STG	42/37		6.58	−52	−42	25
	R PMd	6	255	7.73	26	−10	69
	L IPL	40	246	7.61	−42	−56	57
	L SPL^a^	7		6.19	−38	−58	69
	L angular gyrus	39		5.46	−48	−62	45
	L ITG	37	71	7.26	−60	−56	−7
	R MOG	19	93	7.02	34	−88	33
	L calc gyrus^a^	17	55	5.48	−20	−64	9

*Clusters of proprioception-related brain activation are reported at the cluster-level threshold of *p* < 0.05 FDR corrected*.

*^a^Anatomical locations with more than one maximum. Within each cluster (>50 voxels), only the most significant maximum is listed per anatomical location. BA, Brodmann area; calc gyrus, calcarine gyrus; IPL, inferior parietal lobe; ITG, inferior temporal gyrus; L, left; LWP, left wrist proprioception; MFG, middle frontal gyrus; MOG, middle occipital gyrus; PMd, dorsal premotor cortex; PMv, ventral premotor cortex; R, right; RWP, right wrist proprioception; SI, primary sensory cortex; SMA, supplementary motor area; SMG, supramarginal gyrus; SOG, superior occipital gyrus; SPL, superior parietal lobe; STG, superior temporal gyrus; Sup M Gyr, superior medial gyrus*.

### Laterality of Proprioception-Related Brain Activation

**Figure 5:**
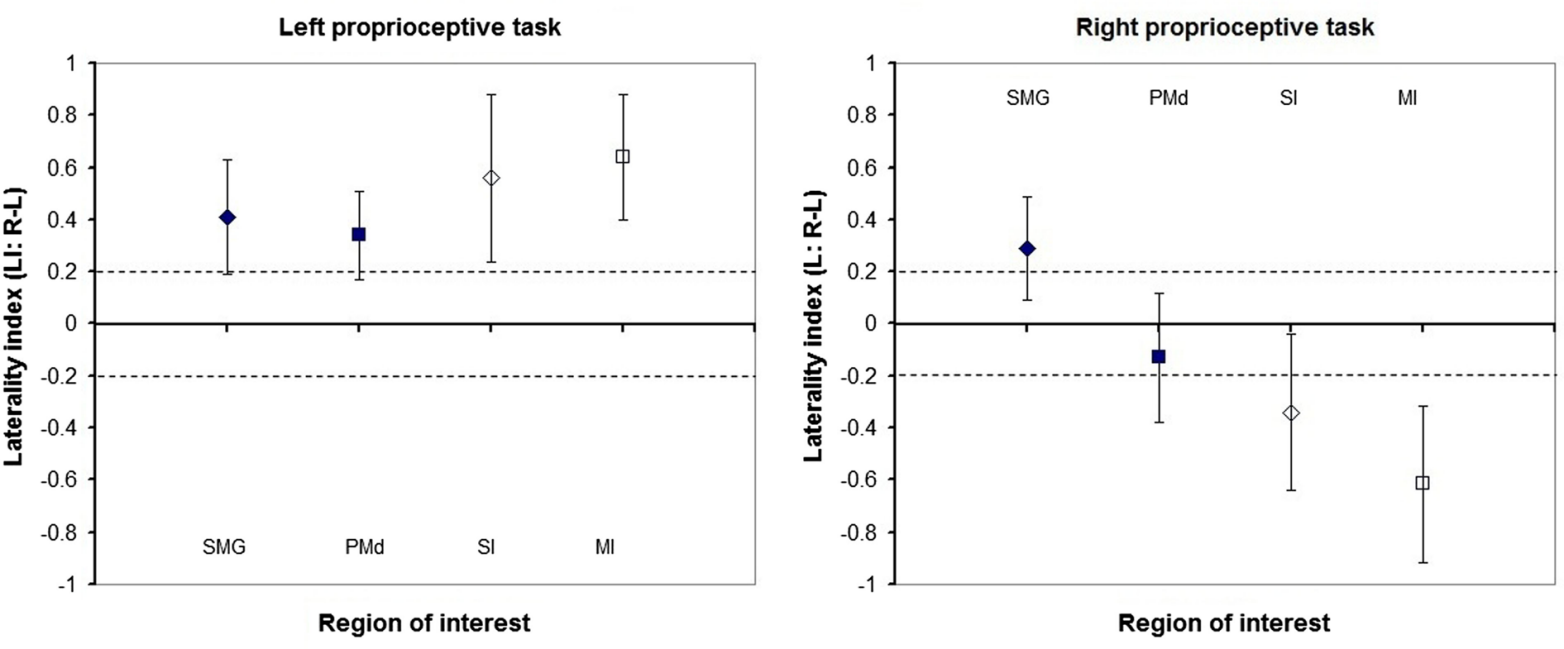
**Laterality of proprioception-related brain activation in regions of interest of healthy participants**. Group mean and standard deviation of laterality indices of the: supramarginal gyrus (SMG), dorsal premotor cortex (PMd), primary somatosensory (SI), and motor (MI) cortices. Diamonds represent sensory cortices and squares motor cortices. Filled shapes represent high-order cortices, while outlined shapes represent primary cortices. Dashed lines represent absolute laterality indices of 0.2. Laterality indices higher than 0.2 represent greater cerebral activation in the right compared to left hemispheres and vice versa for values smaller than −0.2.

Laterality was investigated for the SMG and PMd, high-order somatosensory and motor cortices identified in the group analyses and for the SI and MI given their well-established role in proprioception (see Figure 2). Right laterality of SMG activation was observed for both the LWP and the RWP scans (see Figure 5; Table 3). Laterality calculations for the PMd illustrated a lesser degree of laterality compared to the SMG, with contralateral activation during LWP and bilateral activation during RWP (see Figure 5; Table 3). As expected, LIs of the SI and MI showed contralateral activation (see Figure 5; Table 3). For stroke-affected participants, brain activation was bilateral in both the SMG and PMd (see Table 3).

**Table 3:** **Laterality calculations of brain activation during proprioception of healthy and stroke-affected participants**.

Anatomical region	Healthy				CS1 LWP LI	CS2 RWP LI	CS3 RWP LI
	LWP LI (*n* **=** 12)		RWP LI (*n* **=** 6)				
	
	Mean	SD	Mean	SD
SMG	0.41	0.22	0.29	0.21	−0.18	−0.19	−0.05
PMd	0.34	0.17	−0.13	0.25	−0.06	0.02	0.18
SI	0.56	0.32	−0.34	0.30	−0.56	0.42	0.19
MI	0.64	0.24	−0.62	0.30	−0.77	0.66	0.59

*Positive values indicate right hemisphere activation greater than left and vice versa for negative values. Stroke-affected participants are listed as CS1–3. LI, laterality index; LWP, left wrist proprioception; MI, primary motor cortex; PMd, dorsal premotor cortex; RWP, right wrist proprioception; SI, primary sensory cortex; SMG, supramarginal gyrus*.

## Discussion

We investigated the brain–behavior relationship pertaining to processing of proprioceptive stimuli at the wrist. There are three novel aspects to our study design. First, natural proprioceptive stimuli of passive movements were used, and maximal effort was made to control for confounding tactile and motor stimuli. Participants were required to provide accurate and measurable response to each proprioceptive stimulus both in and outside the scanner. Second, the event-related design with its variable interstimulus intervals enabled temporal isolation of brain activation related to coding proprioception. Third, stroke-affected participants with proprioceptive deficits were studied with respect to the effect of pathology on proprioception-related brain activation.

Our findings indicated that proprioception-related brain activation in high-order somatosensory and motor cortices included the SMG and PMd. The right SMG was activated during both RWP and LWP, and its activity was reduced in the presence of proprioceptive deficits. Proprioception-related brain activation in the PMd was contralateral during LWP and bilateral during RWP. Thus, a certain degree of right PMd laterality was also observed during the central processing of proprioception. These findings confirm right hemispheric dominance in the processing of proprioception, but unlike other studies highlight the key role the right SMG plays in proprioception.

### High-Order Proprioception-Related Brain Activation

The findings from our study suggest that the high-order proprioception-related brain activation of both the SMG and PMd is pivotal for the central processing of proprioception. Several studies have identified proprioception-related brain activation in frontoparietal networks; however, various activation loci were suggested (15, 18, 50–52). Both passive movement and illusory vibration studies identified brain activation in the IPL. Within the IPL, most studies reported proprioception-related brain activation in the parietal operculum (5, 9, 15, 17, 51, 53–55) and only a few reported brain activation in the SMG (6, 18, 56, 57). The SMG is located in the lateral aspect of the IPL whilst the parietal operculum is located medially to the SMG and in the roof of the Sylvain fissure (46). Variability across subjects in the cytoarchitectonic maps of the five areas that occupy the surface SMG has been reported (58) and may have contributed to the variable naming of regions (e.g., parietal operculum compared to SMG) in previous studies. The parietal operculum unlike the SMG is best known for its involvement in the processing of tactile stimuli (59). Tactile stimulation may have accompanied some of the passive movement stimuli in previous studies, for example, from the soles of the feet during ankle dorsiflexion (54, 55). Where tactile stimulation accompanied the proprioceptive stimulation, it is not possible to identify which of the two stimuli generated activation in the parietal operculum.

The SMG is part of the somatosensory association cortex which has a role in interpretation of tactile sensory information as well as in perception of space and limbs location (15, 18). Previous literature suggests that frontoparietal activation in the SMG and PMC may be related to the spatial processing of stimuli around the hand (60) or the recognition of voluntary movement in the human, equivalent of the mirror neuron system (61). Such functions would rely heavily on knowledge of one’s limp position. Indeed Brozzoli et al. (60) showed that the posterior parietal cortex was explicitly responsible for the hand’s position sense.

Brain activation in the SMA is the commonest activation in high-order motor cortices identified in illusory vibration (15, 18) and passive movement (51, 54–56, 62, 63) studies. The SMA has been implicated in processes underlying internally guided movements (i.e., active movements). In comparison, the PMd has been associated with externally guided movements (i.e., passive movements) (64). Given that passive movements are externally imposed, higher activation of PMd than SMA was both expected and found in our study.

Frontal activation in the PMd is important for the processing of proprioception, probably due to the tight coupling between proprioception and its use during movement. Bilateral PMd and right SMG activation was found in a brain imaging study of precision grip but not power grip (65). As proprioception is pivotal for precise motor control (3), it is likely that the frontoparietal brain activation found during precision grip included that of proprioception.

Other lines of research have also found functional association between the SMG and PMd. Anatomical studies in primates showed that proprioceptive information travels to the PMd and that extensive connections exist between the posterior parietal lobe and the PMd (66). In a brain imaging study where healthy participants were required to integrate proprioceptive information into spatial visual or somatic sensory tasks, frontoparietal activation (especially in the right hemisphere) was found (67). Finally, lesion studies indicated that the integrity of the parietal cortex, frontal cortex, and their connections was required for recovery from spatial neglect (68).

### Right Hemispheric Dominance During Proprioception

We found activation of the right SMG during both RWP and LWP, and its activity was reduced in the presence of proprioceptive deficits. Some evidence exists for left laterality of proprioception in the IPL (16, 69). Most of the evidence, however, suggests right hemispheric laterality during proprioception. Illusory vibration studies identified lateralized frontoparietal activation in the right SI (BA 2), middle frontal gyrus (BA 44, 45), parietal operculum, and insula (15, 17), with one study reporting activation in the SMG rather than the parietal operculum (18). In passive movement studies of left and right limbs, right hemispheric laterality was evident in the superior temporal gyrus and the parietal operculum for ankle movements (55) or bilateral IPL and parietal operculum for wrist movements (51). Our findings provide support for right hemispheric laterality but identify the right SMG in particular as a key region activated during proprioception. The lack of brain activation in the parietal operculum is likely due to the effort made in our study to minimize confounding tactile stimulus.

Right SMG activation during proprioception may be explained by the role that this region plays in spatial processing (70). In their important work, Stephan and colleagues (70) used identical visual stimuli to perform a simple reaction time task, a lingual task or a spatial task. They found that despite the common visual stimuli only the spatial processing task activated the right SMG and the junction of the occipital, parietal, and temporal lobes. We regard proprioception as a spatial-processing task because it involves judgments of a limb’s spatial location. If proprioception is a spatial-processing task and the right SMG is a key brain region involved in spatial processing, then this could explain the significance of right SMG activation found in our study.

Studies of participants with hemispatial neglect have also demonstrated an association with right SMG lesions (71). The diagnosis of hemispatial neglect is often made based on visuo-spatial assessment (72), which involves the extrapersonal space. Committeri et al. (73) showed that lesions in the right SMG were particularly related to impaired spatial processing in the personal space studying a large sample of participants with hemispatial neglect, although proprioception as such was not tested. Our findings raise the question of whether hemispatial neglect caused by right SMG lesions not only affects personal space in general but also affects proprioception specifically.

### The Effect of Proprioceptive Deficits Poststroke on the Central Processing of Proprioception

The thalamus was the common lesion site of the three stroke-affected participants included in our study. For two of the participants (CS1 and CS3), the brain lesions extended to the internal capsule, and both displayed more severe proprioceptive deficits on behavioral testing (the Wrist Position Sense Test and the pre­scan behavioral measures). Similar lesion sites in the thalamus and the internal capsule were found in other studies of participants with proprioceptive deficits (74–79).

We found that SMG activation was bilateral in stroke-affected participants. This was the most significant difference observed from the proprioception-related brain activation patterns in healthy participants, where right SMG laterality was found. The findings from stroke-affected individuals with proprioceptive deficits are consistent with the significance of right SMG integrity for adequate proprioceptive function. In previous brain imaging studies of stroke-affected participants where passive movement stimuli were delivered, participants with somatosensory deficits were specifically excluded (50–52, 77, 80, 81). Our findings are therefore not comparable and are novel for stroke survivors with quantified proprioceptive deficits.

Of interest is our finding of ipsilateral brain activation in SIMI. A similar pattern of ipsilateral rather than contralateral SIMI activation has been found in stroke-affected individuals with motor deficits (82, 83). Furthermore, ipsilateral SIMI activation was found in the studies of participants with tactile deficits who performed a touch discrimination task during scans (84, 85). Our findings suggest that similar to other sensory and motor modalities, proprioceptive deficits are associated with a shift of brain activation to the ipsilateral SIMI.

### Study Limitations

Sample size is the main limitation for this study. Twelve participants performed the LWP and only six of them performed the RWP. Due to the smaller RWP group size, group analyses were conducted with a threshold of 0.001 uncorrected for multiple comparisons. Such a threshold increases the risk of false positives, i.e., reporting activation that did not actually occur. To assess the effect of this risk on our results, two additional analyses were conducted. First, group analysis of the LWP was performed at a threshold of 0.05 corrected for multiple comparisons (FDR). Second, a LWP group analysis was conducted for the six participants who also performed the RWP. Results of both analyses showed the same patterns of brain activation were maintained with the same anatomical loci. To minimize the risk of false positives reported in this paper only activation under the threshold of 0.05 corrected at the cluster level was reported. Thus, the additional analyses designed to address limitations related to sample size and threshold, supported the principal proprioception-related brain activation identified in this study.

Contralateral brain activation in SI was not found during RWP. The laterality calculation showed that SI activation during RWP tended to be bilateral. In another brain imaging study of arm proprioception, bilateral SI activation was found during right stimulation compared to contralateral activation during left stimulation (15). In our study, bilateral SI activation during RWP together with the small sample size was the likely cause for activation not reaching significance level. Thus, bilateral SI activation was under represented in our study.

### Clinical Implications

The presence of laterality in proprioception-related brain activation suggests differences in the central processing of proprioception arriving from the left and right limbs. Previous behavioral studies have identified smaller absolute errors for left compared to right limb proprioception (26–28). Our findings together with those of previous brain imaging studies support right hemisphere dominance of proprioception.

Right hemisphere dominance for proprioception has clinical implications for both assessment and treatment. Particular care appears necessary when assessing proprioception in people with brain lesions affecting the right hemisphere, particularly the SMG. The question of which assessment tool to use for proprioceptive assessment is beyond the scope of this paper. However, accurate quantitative tools with normative ranges such as the Wrist Position Sense Test (38) are preferred. A relevant clinical question is the relative contribution of lesions in the right SMG and PMd to proprioceptive deficits.

People with right hemispheric lesions are more likely to require specific proprioceptive rehabilitation. Furthermore, based on the studies of recovery from spatial neglect (68), recovery from proprioceptive deficits may be a function of right SMG and or PMd integrity. A future study examining the relative effect of rehabilitation on right SMG and PMd function would be useful, as would studies on whether normalization of brain activation in these regions correlate with functional recovery.

## Conclusion

We present a novel and innovative brain imaging study of proprioception, where participants were required to provide a direct response to each stimulus, and where response accuracy was monitored. This is the first time that laterality of proprioception-related brain activation has been directly studied with a natural proprioceptive stimulus (passive movements). This is also the first time that such stimuli have been used to examine brain activation in stroke affected individuals with proprioceptive deficits. We achieved temporal isolation of brain activation during coding of proprioceptive stimuli by using the event-related study design. This activation involved high-order somatosensory and motor cortices, namely the SMG and PMd, respectively. Laterality analyses and lesion studies indicated that the right SMG plays a key role in the processing of proprioception. The results provide a novel insight into the brain–behavior system of proprioception and how it is affected by brain lesions. These insights suggest that people with right hemispheric lesions may be more susceptible to proprioceptive deficits, particularly if the right SMG is affected. As the right SMG is commonly implicated in spatial neglect, it raises important questions of whether spatial neglect and proprioceptive deficits are different or associated impairments, and what the relative contribution of the SMG and PMd to proprioceptive function might be. If SMG and PMd lesions affect proprioception differently, then it is possible that different treatment methods may be required to address these differential impairments.

## Author Contributions

EB-S contributed to conception, data collection, analysis, interpretation, and manuscript preparation. TM contributed to conception, interpretation, and critical revision of the manuscript. GP contributed to conception, analysis, and critical revision of the manuscript. AB contributed to conception, analysis, interpretation, and critical revision of the manuscript. LC contributed to conception, data collection, analysis, interpretation, and critical revision of the manuscript.

## Conflict of Interest Statement

The authors declare that the research was conducted in the absence of any commercial or financial relationships that could be construed as a potential conflict of interest.

## Footnotes

^1^http://www.neurobs.com/presentation.

^2^http://www.brain.org.au/software.html.

## Abbreviations

BA, Brodmann area; fMRI, functional magnetic resonance imaging; IPL, inferior parietal lobe; LI, laterality index; LWP, left wrist proprioception; MI, primary motor cortex; PMd, dorsal premotor cortex; RWP, right wrist proprioception; SI, primary somatosensory cortex; SIMI, primary sensorimotor cortex; SII, secondary somatosensory cortex; SMA, supplementary motor area; SMG, supramarginal gyrus.
